# Major swine viral diseases: an Asian perspective after the African swine fever introduction

**DOI:** 10.1186/s40813-020-00159-x

**Published:** 2020-07-06

**Authors:** Roongtham Kedkovid, Chaitawat Sirisereewan, Roongroje Thanawongnuwech

**Affiliations:** 1 Department of Veterinary Medicine, Faculty of Veterinary Science, Chulalongkorn University, Bangkok, 10330 Thailand; 2 Swine Reproduction Research Unit, Chulalongkorn University, Bangkok, 10330 Thailand; 3 Department of Veterinary Pathology, Faculty of Veterinary Science, Chulalongkorn University, Bangkok, 10330 Thailand

**Keywords:** African swine fever, Asia, Pigs, Virus

## Abstract

Asia is a major pig producer of the world, and at present, African swine fever virus (ASFV) continues to significantly impact the Asian pig industry. Since more than 50% of the world’s pig population is in Asia, ASFV outbreaks in Asia will affect the global pig industry. Prior to the introduction of ASF, several outbreaks of major swine viruses occurred in Asia over the last two decades, including porcine reproductive and respiratory syndrome virus (PRRSV), porcine epidemic diarrhea virus (PEDV) and foot and mouth disease virus (FMDV). The rapid spreading of those viruses throughout Asia involve many factors such as the various pig production systems and supply chains ranging from back-yard to intensive industrial farms, animal movement and animal product trading within and among countries, and consumer behaviors. ASF has notoriously been known as a human-driven disease. Travelers and international trading are the major ASFV-carriers for the transboundary transmission and introduction to naïve countries. Globalization puts the entire pig industry at risk for ASF and other infectious diseases arising from Asian countries. Disease control strategies for the various pig production systems in Asia are challenging. In order to ensure future food security in the region and to prevent the deleterious consequences of ASF and other major viral disease outbreaks, disease control strategies and production systems must be improved and modernized.

## Background

Major viral disease outbreaks occur continually in the pig industry. Some diseases can be eradicated or effectively controlled while others become endemic and cause sporadic outbreaks in affected countries. African swine fever (ASF), caused by African swine fever virus (ASFV), is spreading throughout Asia and causing severe economic losses in pig production in several countries. As reported by the World Organization for Animal Health (OIE), affected countries include China, Mongolia, Vietnam, Cambodia, Hong Kong, North Korea, Laos, Myanmar, Philippines, South Korea, Timor-Leste and Indonesia [1].

Asia contributes greatly to the global pig industry and pork production. China alone accounts for almost half of the global pig production. Vietnam, Philippines, South Korea, Japan and Thailand also have large numbers of pigs, though they are mostly for local consumption. The ASF outbreaks contributed to inflation of food prices in many affected Asian countries. The supply shortage and increasing demand of pork will eventually affect global pork prices.

Swine disease control in Asia needs to be enhanced if pig production is to survive in the long term, especially in the era of ASF. In general, disease spread in many Asian countries could be rapid. Small (back-yard) to medium farms with poor biosecurity are among the key factors involved in initial outbreaks in naïve areas. These smallholders tend to feed the pigs with improperly treated/processed leftover food or food-waste (swill feed) from various sources such as households, markets, factories, restaurants, or school canteens, which could contain contaminated pork products. In China and Vietnam, more than 80% of pig farms are small-scale producers with very few large (industrial) farms. Moreover, the back-yard and industrial farms are frequently found in close proximity to each other. This could be the major factor facilitating the faster spread of ASFV in Asian countries when compared with the European countries where wild boars play a major role in disease spread [2, 3].

In addition to back-yard farming, pig and pork movement and trading within and between neighboring countries, as well as consumer behaviors, could also promote disease transmission. The pork supply chains are often quite long, containing multiple stake-holders such as pig farmers, brokers, traders, slaughterhouses, retailers, and consumers [4]. It is common that pig traders, i.e. those who buy pigs from farmers or brokers and sell them to slaughterhouses or other traders, work across long distances, especially when price gaps are high due to the country’s food price inflation. This could facilitate rapid and long-range disease transmission both within and between countries via the movement of infected animals and contaminated vehicles. Local small-scale slaughterhouses and wet markets are also important factors in disease spread [4–6]. Many consumers still prefer buying pork from wet markets found throughout these countries. Poor biosecurity management in small-scale slaughterhouses and wet markets likely causes contamination of pork and its products in the area with various swine pathogens. Therefore, multiple factors, such as back-yard farming, pig/pork movement, consumer behaviors, small-scale slaughterhouses, and wet markets, synergistically contribute to disease transmission in Asian countries.

This review article describes and discusses prior major outbreaks of the swine viral diseases in Asian countries followed by a discussion and elaboration of the consequence of the ASF epizootic in Asia. Finally, an opinion on the future of the Asian swine industry after the ASF outbreak is provided.

### Spread of major viral diseases of swine in the Asian countries

Among the swine viruses that have spread pandemically in Asian countries and caused severe economic losses over the last two decades, porcine reproductive and respiratory syndrome virus (PRRSV), porcine epidemic diarrhea virus (PEDV), foot-and-mouth disease virus (FMDV) and ASFV are inarguably the most notable.

The impact of these outbreaks in China could be economically devastating. This is not unexpected since it has the largest pig population. Increasing numbers of naïve animals in the country, particularly the increased utilization of naïve replacement gilts due to agricultural industrialization, is of utmost concern. Moreover, China seems to be one of the key factors in disease spread.

#### PRRSV

PRRSV is an enveloped virus with single-stranded genomic RNA of approximately 15.4 kb. The virus is classified to the family *Arteriviridae*. There are 10 open reading frames (ORFs) in the PRRSV genome. ORF1 encodes non-structural proteins while ORF2–7 encode structural proteins. PRRSV can be divided into two species including *Beta-arterivirus suid 1* or PRRSV1 (formerly known as ‘European strain’) and *Beta-arterivirus suid 2* or PRRSV2 (formerly known as ‘North American strain’). In general, PRRSV infection in sows results in reproductive failure and infection in growing pigs results in non-fatal respiratory disease, unless complicated with other pathogens [7]. There are three subtypes of PRRSV1 and nine lineages of PRRSV2 (based on ORF5 phylogeny) [8]. Co-circulation of PRRSV1 and PRRSV2 has been reported in several Asian countries including China, Vietnam, and Thailand [9–11].

Overall, the genetics of PRRSV in Asian countries have been dynamic. Independent evolution [12], transmission among Asian countries, introduction of novel PRRSV strains from outside Asia [13, 14] and recombination between PRRSV strains [15] were all involved. Moreover, use of PRRS modified live virus (MLV) vaccines could also impact PRRSV genetic variation and the ongoing genetic changes of PRRSV [16] .

**Fig. 1:**
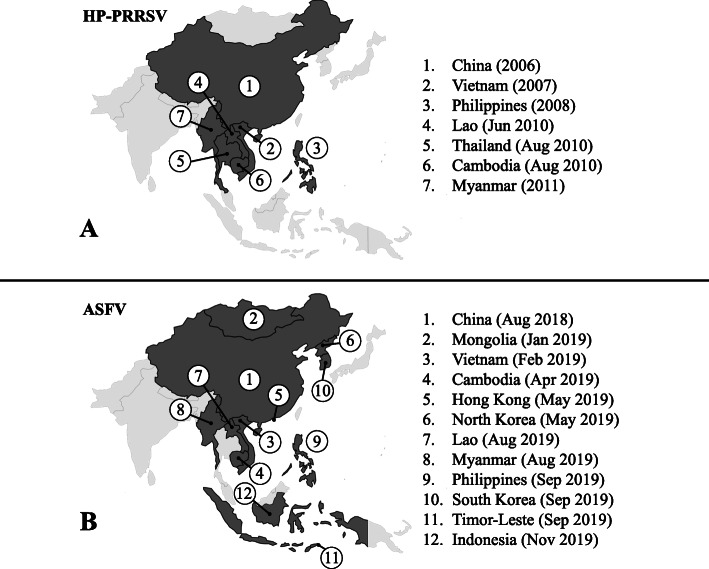
Asian countries with confirmed HP-PRRSV (2006–2011) and ASFV (2018–2019) outbreaks. A number in circle represent chronological order of the first identification of HP-PRRSV (**a**) and ASFV (**b**)

The emergence of a PRRSV2 strain in China in 2006, called ‘highly pathogenic PRRSV’ (HP-PRRSV), could be considered a milestone in PRRSV molecular epidemiology in Asia. Before 2006, PRRSV2 was endemic in many Asian countries. In China, the CH-1a-like strains, belonging to lineage 8, dominated. The CH-1a-like strains are also considered a classical Chinese PRRSV2. Other lineages such as lineage 3 and 5 were also reported [11]. In 2006, HP-PRRSVs e.g. JXA1 strain, were identified which caused the first China HP-PRRS outbreak [17]. Infected pigs exhibited signs of severe, high, hemorrhagic fever and cyanosis, with a high mortality rate. These viruses belong to lineage 8 and are thought to have evolved from the local viruses. Local pork consumption in China had been growing before the outbreak. Many new industrial farms had been established resulting in increased numbers of naïve replacement gilts, which has been speculated to drive mutation of the local viruses. The disease became endemic after the outbreak and the prevalence of HP-PRRSV declined, with only sporadic cases reported in 2008. MLV vaccines based on the Chinese HP-PRRSVs, such as JXA1-R, were also used locally. The second China HP-PRRS outbreak occurred again during 2009–2010. This outbreak was caused by PRRSV2 strains that were genetically different from the 2006 HP-PRRSVs [18, 19]. Since the emergence in 2006, HP-PRRSVs spread and became the dominant strains in many Southeast Asian countries (Fig. 1a), especially Vietnam, the Philippines, Thailand, Cambodia, Laos, and Myanmar. It should be noted that HP-PRRSV outbreaks were first detected outside China in 2007, in Vietnam [20] and then in 2008, in the Philippines [21]. Since the northern part of Vietnam collects pigs for transport to both China and other parts of Vietnam, this could be the route and/or mechanism of HP-PRRSV transmission from China to northern Vietnam followed by spread to the southern part of Vietnam [4, 22]. In 2010, after the second HP-PRRSV outbreaks in China, the virus again spread to Vietnam causing HP-PRRSV re-emergence in the country. Subsequently in 2010, HP-PRRSV outbreaks were reported in Laos (June) [23], Thailand (August) [24], and Cambodia (August) [25]. Due to geographical locations of the outbreaks and virus genetics, it has been speculated that the virus might spread through (illegal) transportation/trading of infected pigs across the borders. The virus may have spread from Vietnam to Laos and Cambodia, and then from Laos it spread further to Thailand. In 2011, HP-PRRSV outbreaks were found in Myanmar [26]. Based on the similarity of the virus genetics, it is suspected that the virus might have spread through pig trading during the Chinese lunar new year at the Chinese southern border. In conclusion, illegal transportation of infected pigs across borders and back-yard farming with poor biosecurity seem to be the key factors in the HP-PRRSV outbreaks from China to the Southeast Asian countries.

After the two HP-PRRSV outbreaks, molecular epidemiology of PRRSV2 in China was complicated by the emergence of various novel PRRSV2 strains, possibly due to multiple introductions, including 1) the emergence of the NAD30-like viruses (lineage 1) in 2012 [27], 2) the emergence of the NADC34-like viruses (lineage 1) in 2018 [14, 28] and 3) the diversified genetics of the lineage 3 viruses [29]. The presence of these novel PRRSVs in Asian countries requires further studies.

#### PEDV

PEDV is an enveloped virus with single stranded genomic RNA of approximately 28 kb. The virus belongs to the family *Coronaviridae*, genus *Alphacoronavirus*. There are seven ORFs in the genome. ORF1a and ORF1b encode nonstructural proteins involved in virus replication. The remaining five ORFs encode structural and an accessory protein including spike (S), ORF3, envelope (E), membrane (M) and nucleocapsid (N) proteins. The virus infects intestinal epithelial cells causing atrophic enteritis. Infected pigs have watery diarrhea, vomiting, and dehydration. High mortality is often observed in affected piglets.

Molecular epidemiology of PEDV is complex due to recombination. For the simplicity of this review, previous proposed nomenclature is used [30]. There are two major strains of PEDV including 1) classical strains, (CV777-like strain) which is the prototypical PEDV and 2) emerging PEDV strains, that have spread globally since 2010. The emerging strains are further classified into two sub-groups including 1) non-S INDEL (insertion and deletion) and 2) S INDEL strains. Both classical and emerging strains could be found during the pandemic in many Asian countries.

The origin of the emerging PEDV strains is not yet known. Before 2005, PED reports in Asia were mostly sporadic cases from China and South Korea. Most of the viruses identified were classical strains. However, severe PEDV outbreaks caused by the non-S INDEL emerging strains were firstly reported in Thailand in 2007 [31], Vietnam in 2009 [32], China in 2010 [33] and then South Korea in 2013 [33]. The virus also spread to many other Asian countries. Although the first report of the non-S INDEL emerging strains in South Korea was in 2013, it was later found that the emerging strains were already present in the country before 2005 [33, 34], suggesting this might be the origin of the PED pandemic. Currently, there is no explanation why severe outbreaks of the emerging strains were not observed in South Korea until 2013. It is possible that complete or partial cross-protection from the endemic classical PEDV might have played a role. It should be noted that large numbers of pigs (one-third of the population) were culled in South Korea during 2010–2011 due to FMDV outbreaks. Whether this mass culling might have also delayed [35] or actually triggered the outbreak of the emerging strains in 2013 is of interest. In 2013, both non-S INDEL and S INDEL strains emerged in the US and the viruses then spread via contaminated fomites to Canada and many European countries. The origin of the S INDEL strains is also currently unknown. They were firstly reported in the US in 2013, however, it was later shown that they could readily be found in China during the outbreak in 2010–2012 [33].

In Southeast Asia, the spreading pattern of non-S INDEL PEDVs is quite different from the HP-PRRSV outbreak. The non-S INDEL PEDV strains might have first emerged in South Korea. However, the route of the transmission into Thailand in 2007, or Vietnam in 2009, is not known since the initial outbreaks in both countries were found in the middle of the countries. It is also possible that non-S INDEL PEDVs had been introduced into China before the major outbreak in 2010. The disease might not have been pronounced due to partial cross-protection from the endemic classical PEDVs. Interestingly, the second HP-PRRSV outbreak in China occurred during 2009–2010. Whether that outbreak might have played a role in the PEDV outbreak in China should be further studied. It could be similar to the suspected association between the FMDV and PEDV outbreaks in South Korea mentioned previously.

PEDV has been endemic in many Asian countries. Re-emerging of PEDV in the infected herds could be a major source of virus. Poor management of gut feedback, gilt acclimatization and biosecurity could probably be key factors for virus circulation in the same herds [36]. Effective PED vaccines are needed for sow herd stabilization in endemic farms.

#### FMDV

FMDV is a non-enveloped virus with single stranded RNA genome of approximately 8.4 kb. The virus belongs to the *Picornaviridae* family, *Aphthovirus* genus. The RNA genome contains a single ORF encoding a polyprotein that undergoes proteolytic processing to give rise to four structural proteins (VP1 – VP4) and nine non-structural proteins (2A, 2B, 2C, 3A, 3B1, 3B2, 3B3, 3Cpro and 3Dpol). There are seven serotypes, including O, A, C, Asia-1, Southern African Territories (SAT) 1, SAT2 and SAT3. Cloven-hoofed animals such as pigs, cattle, sheep, goats and buffaloes are susceptible to FMDV infection. Disease transmission between different species is possible. Virus transmission in pigs occurs mainly via the oropharyngeal route. The major clinical signs include vesicular lesions at the snout, tongue, and the coronary bands and heel bulbs of the feet. The virus can also infect the myocardium of neonatal piglets, resulting in myocarditis. The mortality of FMD in adult pigs is generally low. However, high mortality can be observed in the FMD-related myocarditis cases [37]. For inter-species transmissions, pig often act as an amplifying host by producing large amounts of virus that are aerosolized and further infect other animals [38].

FMD endemic regions are classified into seven pools (1–7) based on the shared viruses circulating in each geographical area [39]. There are three FMDV pools in Asia including *pool 1* (Southeast Asia and East Asia), *2* (Southern Asia) and *3* (Middle East and West Eurasia).

Animal movement, vaccination policy, and farm biosecurity seem to be the major factors affecting FMDV endemicity and transmission both within and between FMDV pools in Asia. Cross-border cattle transmission can be found in many neighboring countries. Large numbers of cattle are located in India (FMDV *pool 2*) which move in various directions, such as westward to Pakistan, northward to Nepal, and eastward to Bangladesh. The eastward cattle movement from India has the potential to eventually reach Southeast Asian countries (FMDV *pool 1*) [39]. Starting from 2015, the recent spread of FMDV O/ME-SA/Ind-2001 from India to both cattle and pigs in many regions, including Southeast Asia, [40, 41] might be supported by this animal movement pattern. Apart from animal movement, vaccine coverage might also be another important factor disease spread. In mainland Southeast Asia, FMD vaccination is generally limited to cattle, and only in certain regions with high risk of infection [42]; FMD vaccination in Thailand is done in both cattle and pigs across the country. Moreover, large numbers of cattle in Southeast Asia are on small farms with poor biosecurity management, thereby contributing to virus transmission in the area. Taken together, FMD control could be challenging in many Asian countries due to uncontrolled animal movement.

Recently, Seneca valley virus (SVV) has been identified in Asia, including China [43] and Thailand [44]. Diagnosis of vesicular disease outbreaks should then be made with caution, especially in FMD-endemic countries since both SVV and FMDV produce similar vesicular lesions in infected pigs.

#### ASFV

ASFV is an enveloped virus with genomic DNA of approximately 170–193 kb. The virus is a member of the family *Asfarviridae*. The genome of ASFV encodes many proteins. Based on the partial nucleotide sequence of the capsid gene, the virus can be classified into at least 24 genotypes. Infected pigs show various clinical signs ranging from sudden death and acute hemorrhagic fever to chronic infection. In general, ASFV spread in swine herds requires close contact between animals and not airborne. Mortality in infected animals often reaches 100% [6, 45].

Compared with PRRSV and PEDV outbreaks, molecular epidemiology of ASFV is less complex. Only the genotype II viruses are responsible for the current ASF outbreaks in Asian and European countries. The virus spread from Africa to Georgia in 2007, with further spread to many European countries [46]. The virus was then found in Russia and finally reached China in 2018 [47]. The virus then spread to many Asian countries (Fig. 1b) and only a very few, such as Thailand and Taiwan, are still negative [1].

ASFV transmission occurs via direct and indirect contact. Moreover, the virus has been shown to be highly resistant in the environment [48], and may persist for long periods of time in contaminated fomites or pork products, making environmental disinfection challenging. When infected blood is involved, disinfection is especially critical because blood contains higher levels of infectious virus compared to virus excretion levels in oral and nasal fluids and feces [49]. A recent study has shown that although ASFV in excretion can be effectively transmitted to naïve pigs via an indirect route, there is only a short time period for this transmission to occur [50]. Therefore, carcass and waste management of farms in affected areas could be extremely vital to disease eradiation and control. ASF in European and Asian countries is quite different. The European situation mostly involves outbreaks in wild boars (with the exception of Romania), while the Asian situation is oriented on domestic swine [6]. ASF in European countries is generally considered a human-driven disease where it is found far away from initial outbreaks. This is also true for the situation in Asian countries. It has been suspected that the outbreak in the affected Asian countries might start from back-yard farms after infected pork products were brought into the region through the human-food chain and finally reached the backyard farms via swill feeding. Once the infection is established on the farm, the virus can be transmitted by contaminated fomites through the local supply chains to other farms, especially those with poor biosecurity. Emergency sale of infected pigs could also play an important role in the virus spreading and spilling over to other areas and countries. The viral load in the environment or vehicles in the affected areas might then increase due to virus transmission among small holders and other pig supply chains. At that point, even industrial farms with high health status and good biosecurity might also be at risk.

Currently, there are still some ASFV-negative Asian countries/regions including Thailand and Taiwan. This could be due to effective collaboration among stake holders including farmers, private sectors, academia, and government authorities. In general, farmers must be extremely aware of the difficulty and importance of disease prevention and control of ASF when introduced. Strict border control is required to prevent virus introduction, especially from contaminated vehicles and pork products, at all ports of entry. *Restricting* imports of *pork* and *pork products* from affected countries are also necessary. The number of unqualified back-yard farms must be reduced, especially at the border regions, which is critical to prevent ASF contamination of the environment. Key factors to success in keeping the disease out of each country might vary. For example, in Thailand, which is surrounded by ASF-positive countries, back-yard farms are mostly located in rural areas, especially at the country’s border area. Most industrial farms are located in the remoted areas far away from the community to avoid the conflict with the community [6]. Therefore, border security has been highly strengthened to prevent virus introduction from the neighboring countries, e.g. cleaning facilities for disinfecting pig transport vehicles have been installed and utilized at various land ports of entry. In addition, the back-yard farms, especially at the border areas, are being routinely monitored for ASF by collaborations of local government officials and private sectors, particularly with volunteering villagers. For Taiwan, which is an island, the prevention measures have focused mainly at the airports and ports to prevent the virus introduction via contaminated pork products illegally brought in by travelers [51]. First-time offenders illegally importing meat products into Taiwan from areas affected by ASF within the past 3 years are fined $6700 with the penalty increasing to $33,500 for repeat offenders and fines are imposed even for bringing in simple snack items such as pork jerky. Monitoring along the sea shore is also required since many infected, dead pigs were found floating from the mainland. Moreover, authorities ordered a complete stop to swill feeding.

### Asian ASF aftermath: a possible threat to global swine production

ASF is currently spreading across many parts of the world, including Asia. The direct impact of the disease to the pig industry is vital and obvious. The disease will probably become endemic in Asia. Recently, much more knowledge on ASFV has been gained, especially now that candidate vaccines with promising results in both efficacy and safety have recently been reported. Sooner or later, ASF control might not be out of the question.

Besides ASFV, PRRSV, PEDV and FMDV, many other swine viruses are circulating in Asia, including both newly characterized and endemically well-known viruses. In addition to these four major viruses, it is interesting whether other swine viruses might soon play an important role in the global pig industry, particularly if those viruses spread to the other major pig producing regions such as North America and Europe.

In the past decade, various novel swine viruses have been identified around the world. Since these viruses are newly discovered, some important information might not be clearly understood in term of epidemiology, pathogenesis, and disease diagnosis. Fortunately, newly identified viruses are not necessarily new as they are often related to a known virus, nor will they always result in outbreaks.

Porcine circovirus 3 (PCV3), for example, was firstly reported in the US in 2015 [52, 53]. PCV3 pathogenesis is still not fully understood. Soon after the discovery, identification of PCV3 was published in many regions, including Asian countries such as China [54], South Korea [55], and Thailand [56]. It was later shown that the virus had existed endemically in the global pig population for a very long time before the first report [57, 58]. This endemic status might partly protect the population from the severe losses since some animals already have immunity against the virus. Still, naïve populations might be prone to the outbreaks.

In contrast to the PCV3 situation, viruses only reported in Asia are more threatening to the pig industry. Recently, a new alphacoronavirus causing fatal diarrhea in piglets has been reported in China. The virus was named swine acute diarrhea syndrome (SADS-CoV), a.k.a. swine enteric alphacoronavirus (SeACoV) and porcine enteric alphacoronavirus (PEAV). The spread of SADS-CoV, if it occurs, might possibly be as devastating as the PEDV outbreak since the two viruses share some similarities. They both belong to the genus *Alphacoronavirus* of the family *Coronaviridae* and cause fatal diarrhea in piglets, especially those less than 1 week old. Interestingly, Asian countries might play a key role for both viruses to spread world-wide. The 2010–2013 PEDV pandemic started in Asia and SADS-CoV has recently been discovered in China. Although the spread of SADS-CoV has been very limited compared to PEDV, it should not be overlooked. SADS-CoV was discovered in China in February 2017 [59] causing outbreaks in at least four farms in the Guangdong Province of Southern China resulting in the death of approximately 25,000 piglets. The disease seemed to disappear 4–5 months after the start of the outbreak. This could be due in part to the implementation of gut feedback strategies; however, reemergence of SADS-CoV occurred in February 2019 [60], also in Guangdong Province. SADS-CoV was identified on a farm in which approximately 2000 suckling piglets died from acute diarrhea. The results showed that the virus is still circulating in China, at least in some regions. Therefore, there is still a risk of spreading the disease to other countries.

Compared with the novel viruses, well-known viruses may not seem to be as detrimental to the global pig industry at first. Diagnostic protocols, detection methods, or even effective vaccines are often commercially available. However, it should be kept in mind that pigs in some countries or regions might be immunologically naïve. This is especially true when the diseases have already been eradicated and the vaccines were no longer available or used. The viruses will spread rapidly in the naïve population and the outbreak will be devastating.

One recent reemergence of a well-known viral disease is classical swine fever (CSF), caused by classical swine fever virus (CSFV), occurred in Japan in 2018. The disease had long been eradicated in the country, vaccination had been banned since 2006, and the country received a CSF-free status by the OIE in 2007. However, in September 2018, CSFV infection was confirmed in a pig farm [61]. Despite implementation of various CSF control measures, the outbreak did not cease and continued to spread. At the end of November 2019, approximately a year after the reemergence, approximately 120,000 pigs were culled or died. CSFV was also identified in wild boars during the outbreak and virus transmission between wild boars and domestic pigs has been suggested [62], rendering outbreak and disease control extremely challenging.

Highly virulent virus strains are not the only threat for immunologically naïve populations. Even strains with considerably low virulence could be formidable, including MLV vaccines. One of the recent and still ongoing cases is the CSF situation in Jeju Island, South Korea [63]. While sporadic outbreaks have occurred in mainland South Korea, Jeju Island successfully eradicated the disease and became CSF-free in 1999. CSF vaccination was banned in Jeju Island in 1998; in 2014, the virus reemerged. Reproductive failure in sows and cyanosis, diarrhea and death in young pigs were observed. It was later confirmed that the CSF MLV was the causative agent. Epidemiological investigations showed that the CSF MLV strain (Low virulence strain of Miyagi, LOM) was accidentally introduced to Jeju Island through a combined lived vaccine (CSF-swine erysipelas). LOM-related CSFV has currently been wide spreading and endemic in Jeju Island since the reemergence in 2014. The CSF outbreaks in Jeju Island might be a special situation, partly because the LOM MLV itself is still considered virulent, especially in naïve pregnant sows. However, when taking mutation and reversion to virulence into consideration, MLV vaccines of any other viruses might not be completely safe and should be avoided in the naïve populations.

Many Asian countries have been affected economically following the ASF outbreaks beginning in late 2018. Various control measures have been used including pig culling with compensation and restocking with strict biosecurity. Currently, approximately 1.7 million pigs in Asian countries were culled or died from ASF [1] resulting in pork shortage and rising pork prices. Consequently, pig repopulation has been attempted in many ASF-affected countries. Unfortunately, large-scale culling and repopulation with high numbers of naïve replacement gilts can greatly impact the epidemiology and susceptibility to other swine viruses in this region.

The risk that new viruses or viral strains will be introduced into Asia should be considered due to the increasing importation of the breeding pigs for repopulation purpose, e.g. the importation of breeding pigs from European countries to China after the ASF outbreaks. If these imported pigs are not carefully monitored, foreign viruses and other pathogens could be introduced into the countries. The viruses might then be established and even become the dominant circulating viruses causing complicated problems. Moreover, considering that there are already local viruses circulating in Asia, Asian countries could then act as a focus for viral recombination, especially for RNA viruses.

Recombination between novel and local strains is not unexpected when co-circulated within the same farm. In fact, these phenomena have already been shown for some viruses in Asian countries, such as the NADC30-like PRRSVs in China.

The emergence of the NADC30-like PRRSVs has dramatically changed the molecular epidemiology of PRRSV2 in China. HP-PRRSV emerged in China in 2006, and then became the dominant PRRSV2 strains of the country. Due to many factors, HP-PRRSV prevalence then declined. In 2012, the NAD30-like PRRSVs emerged in China and spread throughout the country [27]. The virus then became the dominant PRRSV2 strain, replacing the HP-PRRSVs. It is suspected that imported breeding pigs from the US were the source of the virus introduction. It was also shown later that NAD30-like PRRSVs underwent recombination with various co-circulating PRRSV2 strains in China including the classical Chinese PRRSV2, the previous dominant HP-PRRSVs [15, 64, 65] and even the MLV-related strains such as RespPRRS MLV [66] and the Chinese HP-PRRSV JXA1-R based MLV [16].

Rapid and intense repopulation of naïve animals following the ASF mass-culling might impair the herd immunity against the local viruses as well, if not cautious (e.g. poor biosecurity and gilt acclimation). Genetic bottleneck and founder effects might be involved in mutation of the local viruses in this situation, resulting in establishment of novel strains. Moreover, the increasing naïve animals from repopulation might, in turn, provide more susceptible hosts for the local viruses. Viral load in the area might consequently increase, raising the risk of virus contamination into multiple types of vectors or carriers such as feed ingredients, meat, and pork products. Therefore, the risk that all the types of viruses mentioned previously, e.g. locally endemic viruses, newly identified viruses, recombinant viruses, will spread from Asia to other regions, jeopardizing the global pig industry.

Virus spread from Asia to North America and Europe could be devastating due to the valuable pork and pig breeding industries in those regions. Viruses that have already been eradicated or not existing in those regions, such as the CSF-free status of the US, might require extra precaution since the pig population is immunologically naïve and extremely susceptible. Travelers, feed ingredients, meat and pork products could play a major role in the transboundary transmission. The current situation of ASF has shown that meat and pork products brought by travelers and passengers, especially from the affected countries, is a vital method of spread [67, 68]. This could apply to other viruses as well. Preventing virus transmission via contaminated animal feed ingredients is another serious issue. Countries importing a large amount of animal feed materials from the affected countries are at high risk. It has been shown that various viruses can survive in the feed materials during trans-Pacific and trans-Atlantic transportation models simulating trips from Asian and European countries to the US, respectively [69].

The ASF outbreaks in Asian countries may also have a positive side for the Asian pig industry. The number of high-health status herds might increase while the back-yard farms could be eliminated and eventually disappear. This could be beneficial for producers wanting to survive ASF in the affected countries. Strict biosecurity management and collaboration among all stakeholders of the pork supply chains are required. Farms with poor biosecurity, such as backyard farms, are not able to prevent and withstand the disease. Therefore, if affected countries could endure the outbreaks by having good biosecurity practice, the overall production might rebound and subsequently increase.

In summary, after the introduction of ASFV, Asia might act as a hub for global swine viruses, particularly ASFV and other emerging and re-emerging viruses. Local and foreign viruses/viral strains might be introduced, co-mingled, mutate and then spread to other regions. The Asian pig industry might therefore impact the global pig market.

### The future of the Asian swine industry

With the current ASF situation in Asia and the fact that the global pig industry might consequently be affected, whether by ASFV itself or other viruses from Asia, the future of Asian pig industry could be in jeopardy. Therefore, to prevent detrimental outcomes, strategies used to survive the ASF outbreaks are extremely important. Guidelines and suggestions for developing ASF control strategies have been reported [6]. Now that the disease is already spreading in many Asian countries, the only exceptions being Thailand and Taiwan, some key issues in ASF control in Asia are identified and discussed in this section.

Firstly, small farms using swill feeding and poor biosecurity should be forbidden or closely monitored. Those small farms could be a very important starting point of ASF outbreaks in the naïve areas. The routes of ASFV transmission into these farms could be varied and include swill feeding or contaminated fomites and vehicles. For larger farms with good biosecurity management, the risk of ASF introduction is generally low. However, the risk will be increased when the viral load contaminating the environment in the region increases due to outbreaks on neighboring farms, particularly back-yard farms. Therefore, farm standardization policy to eliminate the farms with poor biosecurity as well as compartmentalization or zoning are highly suggested in the affected countries.

ASF vaccines together with good biosecurity practice will be the key for future pig production. Recently, live ASFV vaccine candidates showing promising results in both safety and efficacy aspects have been reported, such as the ASFV-G-∆I177L virus, the 2007 Georgia isolate with genetically engineered deletion of I177L gene [70] and the HLJ/18-7GD virus, the 2018 China isolate with genetically engineered deletion of seven genes including MGF505-1R, MGF505-2R, MGF505-3R, MGF360-12 L, MGF360-13 L, MGF360-14 L, and CD2v gene [71]. Due to the gene-deleted characteristic of these viruses, they could also serve as marker vaccines which will be extremely valuable in disease control programs such as DIVA (differentiating infected from vaccinated animals) strategies. One thing to keep in mind is that focusing only on the vaccination while neglect other necessary control measures such as biosecurity and animal movement restriction could be problematic. This has been shown previously for other diseases such as pseudorabies virus (PRV) and CSF controls in China [72, 73]. For PRV, marker vaccines are available and highly effective. Therefore, PRV vaccines could greatly support eradication strategies. However, it has been reported that many farmers in China rely mainly on the vaccination, while serological and virological surveillance are rarely implemented. Consequently, infected pigs are not screened and culled from the herds and endemic virus could still spread [73]. Interestingly, it has been shown recently that PRV variants with reduced vaccine cross reactivity were identified in China [74, 75]. Further studies are required to determine whether improper control strategies focusing only on vaccination are involved in the emergence of the PRV variants. Moreover, the number of non-standardized vaccine production units/facilities might rise subsequent to increased vaccine demand. Vaccines with questionable safety and efficacy might then be distributed and cause further problems, as in the case of CSF in China [72].

Prevention measures against the virus introduction into farms through commercial pig feeds or feed ingredients should be further studied. This is one of the most important obstacles in biosecurity management of even large industrial pig farms. This route of transmission has been speculated in many ASF outbreaks in large farms with very strict biosecurity. It has been shown previously that multiple swine viruses could survive in certain feed ingredients for a very long time [69, 76, 77]. For example, one study has shown that the half-life of ASFV in various feed or feed ingredients ranged from 9.6 days (conventional soybean meal) to 14.2 days (complete feed). However, the study used temperature (12.3 ± 4.7 °C) and humidity (74.1 ± 19.2%) that simulated a trans-Atlantic shipment [76]. The results could be different for domestic transportation within and among Asian countries. Recently, key principles to reduce the risk of virus transmission in pig feed has been reviewed [78]. There are some interesting concepts and strategies that might be used for reducing the infectivity of viruses contaminating in the feed, such as heat treatment e.g. pelleting the feed using higher temperature; chemical mitigation e.g. treating the feed with a formaldehyde and propionic acid solution; and storage period management e.g. using the knowledge on the half-life of the virus to adjust the feed storage time and conditions. Whether those strategies are effective or practical for various swine viruses should be further elucidated.

Perception of risk management of farmers and other stakeholders could be fundamental to the disease controls. This could not be stressed more. Many Asian countries have already passed the peak of severe ASF outbreaks and are currently in the endemic stage. Pork prices are now rising. Increasing pig production could be very enticing; however, farmers and other sectors in pig supply chains must not neglect the impact of ASF. Biosecurity management must not be compromised. Continuing education of farmers and other stakeholders in the supply chains is required to prevent ASF reemergence.

Collaboration is another critical factor for the future of Asian pig industry. Disease eradication and prevention on the farm might not be successful if the diseases are still active on neighboring farms. This might also be applied to the international level. Collaboration among neighboring countries should be strengthened. Transboundary disease transmission through smuggled pigs and pork products has been suspected in many diseases in Asia such as the transmission of certain strains of PRRSV into Thailand [24] and the detection of ASFV in pork products brought to Taiwan by travelers [51]. It should be emphasized that the transboundary transmission can occur in geographically isolated regions such as the recent introduction of CSFV into Japan, or in extremely long distance such as the transmission of PEDV from Asia to the North American continent via contaminated fomites. For ASF, all persons involved in pig production should remember that if the problem in Africa is not solved or addressed, it will eventually reappear out of Africa again. Therefore, the concept of collaboration might extend beyond neighboring countries via Food and Agriculture Organization (FAO) networking.

In late 2019, a novel human respiratory disease called coronavirus disease 2019 (COVID-19) caused by severe acute respiratory syndrome coronavirus 2 (SARS-CoV-2) emerged. The disease was first identified in Wuhan, China. Currently, COVID-19 spread pandemically affecting people in many countries [79]. The effect of COVID-19 on the future swine industry is not yet elucidated. SARS-CoV-2 might not infect or cause disease in pigs. However, the COVID-19 pandemic can indirectly effect pork production. For example, social distancing, mandatory mass-quarantine, and mandatory self-quarantine of workers in pork supply chains due to COVID-19 could interrupt the pig supply chain and pork business, as has already been evidenced in the U.S. COVID-19 outbreaks in the meat processing plants led to closures of some factories and posed a significant threat to the meat supply in the country in addition to huge economic losses for processors and farmers. Millions of farm animals were culled as a result of the meat plant closures. Therefore, in the future, use of machines and implementation of automation should be considered. Ironically, it should be noted that COVID-19 situation may have a positive effect in reducing the ASF spreading between countries due to the lockdown policy and international travel restrictions.

## Conclusion

The Asian pig industry is playing an important role on shaping up the future of global pig production. ASFV could become endemic in Asia for many years to come and the risk of spreading to other regions is possible. In addition to ASFV, there might be other viruses from Asian countries that could be harmful to the global pig industries or vice versa. Therefore, disease control strategies in Asia should be ameliorated to prevent deleterious consequences. Collaboration and information sharing among countries are needed for successful prevention and control of major swine viral diseases.

## Data Availability

Not applicable.

## Abbreviations

ASFV: African swine fever virus
CSFV: Classical swine fever virus
FAO: Food and Agriculture Organization of the United Nation
FMDV: Foot-and-mouth disease virus
HP-PRRSV: Highly-pathogenic porcine reproductive and respiratory syndrome virus
INDEL: Insertion and deletion
LOM: Low virulence strain of Miyagi
MLV: Modified live virus
OIE: World Organisation for Animal Health
ORF: Open reading frame
PCV: Porcine circovirus
PEAV: Porcine enteric alphacoronavirus
PEDV: Porcine epidemic diarrhea virus
PRRSV: Porcine reproductive and respiratory syndrome virus
PRV: Pseudorabies virus
SADS-CoV: Swine acute diarrhea syndrome coronavirus
SeACoV: Swine enteric alphacoronavirus
SVV: Seneca valley virus
